# The effect of intraocular and intracranial pressure on retinal structure and function in rats

**DOI:** 10.14814/phy2.12507

**Published:** 2015-08-19

**Authors:** Da Zhao, Zheng He, Algis J Vingrys, Bang V Bui, Christine T O Nguyen

**Affiliations:** Department of Optometry and Vision Sciences, University of MelbourneParkville, Victoria, Australia

**Keywords:** Electroretinogram, optic nerve pressure gradient, optical coherence tomography

## Abstract

An increasing number of studies indicate that the optic nerve head of the eye is sensitive not only to changes in intraocular pressure (IOP), but also to intracranial pressure (ICP). This study examines changes to optic nerve and retinal structure in a rat model in response to a range of IOP and ICP levels using optical coherence tomography. Furthermore, we examine the functional sequelae of these structural changes by quantifying the effect of pressure changes on the electroretinogram. IOP elevation (10–90 mmHg) induces progressive deformation of the optic nerve head and retinal surface (*P* < 0.05), compression of the retina (*P* < 0.05) and bipolar cell (b-wave), and retinal ganglion cell (scotopic threshold response) dysfunction (*P* < 0.05). Simultaneously altering ICP (−5 to 30 mmHg) modifies these IOP-induced responses, with lower ICP (−5 mmHg) exacerbating and higher ICP (15–30 mmHg) ameliorating structural and functional deficits. Thus, the balance between IOP and ICP (optic nerve pressure gradient, ONPG = IOP − ICP) plays an important role in optic nerve integrity. Structural and functional parameters exhibit a two-phase relationship to ONPG, with structural changes being more sensitive to ONPG modification (threshold = −0.6 to 11.3 mmHg) compared with functional changes (threshold = 49.7–54.6 mmHg). These findings have implications for diseases including glaucoma, intracranial hypertension, and long-term exposure to microgravity.

## Introduction

The optic nerve head is an area which is particularly susceptible to pressure stress. Two key pressure forces act here, intraocular pressure (IOP) and intracranial pressure (ICP). Elevated IOP has been long-established to induce detrimental effects to retinal function and more recently increasing evidence indicates ICP may also play an important role [see Morgan et al. (2015) and Jonas et al. for a comprehensive review (2013)]. The optic nerve is particularly susceptible to pressure, because at this location the sclera thins to form sieve-like layers of collagen allowing retinal ganglion cell axons to exit the eye. As such, these areas along with the peripapillary sclera suffer the highest levels of stress and strain (Downs et al. 2008) and are prone to deformation. Thus, the health of the ganglion cell axons is influenced by pressure inside (IOP) and outside (ICP) the eye on either side of the optic nerve. What has not yet been established is how these biomechanical changes relate to retinal function over a range of IOP and ICP levels. Elucidating this relationship will have important implications for conditions involving abnormal IOP and/or ICP. These include glaucoma (elevated IOP, perhaps decreased ICP), traumatic brain injury (elevated ICP), and long-term exposure to microgravity-associated visual impairment (elevated ICP, elevated IOP) (Mader et al. 2011; Woo et al. 2012).

Cerebrospinal fluid fills the subarachnoid space that surrounds the optic nerve all the way up to its entry into the eye (Jonas et al. 2003). As such, ICP can influence the biomechanics of the lamina cribrosa and peripapillary sclera. Morgan and co-workers (2002) show in canine eyes that posterior deformation of the optic nerve surface can be induced by IOP elevation, whereas anterior deformation can be produced with ICP elevation. They show that the pressure difference between IOP and ICP, the translaminar pressure gradient, has a critical bearing on the position of the lamina and the topography of the optic nerve head (Morgan et al. 2002). A recent study in nonhuman primates shows that posterior deformation of the optic nerve head and peripapillary tissue manifest following 12 months of mild chronic ICP reduction (Yang et al. 2014). Thus, a higher translaminar pressure gradient due to ICP reduction promotes posterior deformation of the optic nerve surface, which may promote axonal injury. As the current study examines the balance between IOP and ICP in a rodent model which exhibits a rudimentary lamina cribrosa (Morrison et al. 2005), the pressure gradient across the tissue will be termed the optic nerve pressure gradient (ONPG = IOP − ICP). In support of the importance of ICP in glaucoma development, a handful of studies have reported that ICP is reduced in glaucoma patients which results in higher translaminar pressure gradients (Berdahl et al. 2008a; Ren et al. 2010, 2011a,b; Wang et al. 2012). Furthermore, multiple review/opinion articles discuss the role of ICP in glaucoma, illustrating the interest and impact that it is having on the field (Pasquale 2008; Berdahl and Allingham 2010; Wostyn et al. 2011; Jonas et al. 2013; Siaudvytyte et al. 2015).

Whilst the pressure gradient across the optic nerve is known to influence the topography of the optic nerve surface, it is not known to what extent tissue deformation influences retinal function, if at all. This study tests the hypothesis that the retinal structure and function is influenced by ONPG either by way of IOP or ICP modulation, that is, increased posterior deformation of the optic nerve exacerbates retinal dysfunction. We consider this hypothesis in the rat eye, which has an optic nerve structure including a rudimentary lamina cribrosa that provides a useful preclinical model of optic nerve physiology and vascular regulation (He et al. 2013; Lim et al. 2014).

## Materials and Methods

### Subjects

All experimental procedures were in compliance with the National Health and Medical Research Council Australian Code of Practice for the care and use of animals for scientific purposes. Prior to commencement, animal ethics approval was obtained from the Howard Florey Institute Animal Experimentation Ethics Committee (13-044-UM).

Adult male Long-Evans rats (*n* = 5–9 each group, 11–30 weeks, 270–450 g, Melbourne Brain Centre, Kenneth Myer Building, Parkville, Victoria, Australia) were employed in this study. Animals were housed in well-ventilated cages, with access to normal rat chow and water ad libitum. Room temperature was maintained at 21°C and the light cycle was a 12-h light (50 lux), 12-h dark cycle with the lights switched on at 8 am. All experiments were conducted under a ketamine:xylazine anesthesia (60:5 mg/kg, intramuscular, Troy Laboratory, Glendenning, NSW, Australia). Body temperature was maintained at 37.5 ± 0.5°C, using a circulating water heat pad. For all experiments, topical administration of Ophthetic (0.5% proxymetacaine, Alcon Laboratories, Sydney, NSW, Australia), and Mydriacyl (0.5% tropicamide, Alcon Laboratories) provided corneal anesthesia and mydriasis, respectively. Additionally, during ocular coherence tomography (OCT) measurements, Genteal gel (0.3% hypromellose, Alcon Laboratories) was applied to moisten the cornea and to act as an optical coupling medium between the camera objective lens and the eye. For electroretinogram (ERG) recordings, rats were dark-adapted overnight (12 h) (Behn et al. 2003). In addition, care was taken to minimize light exposure during ERG set-up to ensure maximum retinal sensitivity and optimize ganglion cell-specific scotopic threshold response (STR) measurements (Bui and Fortune 2004).

**Figure 1:**
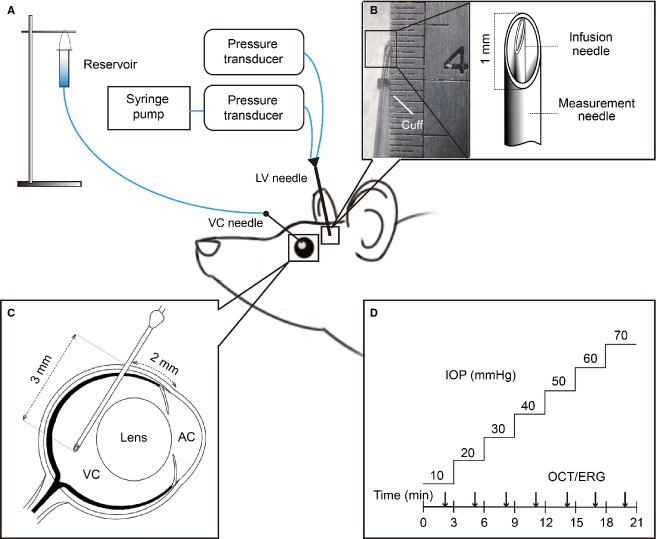
Intraocular and intracranial pressure elevation methodology. (A) IOP and ICP elevation was achieved by placing a needle into the vitreous chamber (VC) and a dual-lumen needle into the ipsilateral lateral ventricle (LV), respectively. A saline reservoir was connected to the vitreous chamber needle, whereas a syringe pump was connected to the lateral ventricle cannula. (B) A custom made dual-lumen needle with infusion (inner needle) and measurement ports (outer needle) was used for ICP elevation. (C) Vitreous chamber cannulation employed a 27G needle inserted 2 mm behind the limbus at a 45 degree angle. (D) At each ICP level, IOP was elevated from 10 to 70 mmHg in 10 mmHg steps each lasting 3 min. Optical coherence tomography (OCT) or electroretinography (ERG) was assayed at each IOP and ICP level.

To determine the effect that ONPG modification exerts on retinal structure and function, an IOP step protocol was employed (He et al. 2006, 2008). This approach allows a wide range of IOP levels to be systematically studied in a short time. The step protocol involves progressive IOP elevation from 10 to 70 mmHg in 10 mmHg steps, with each step lasting 3 min (Fig.1D). OCT or ERG assessment was conducted at each IOP step. An additional IOP step of 90 mmHg was conducted in ERG recordings to induce complete functional loss. Each animal underwent the IOP step protocol twice at two randomly chosen ICP levels (−5, 5, 15, 25 or 30 mmHg). Each IOP/ICP run was separated by 21 min, which was shown in pilot studies to provide sufficient time for functional and structural recovery from a preceding sequence of IOP elevation steps. OCT and ERG measurements were conducted in two parallel cohorts of animals (*n* = 6–7, OCT; *n* = 5–9, ERG).

### Intraocular pressure (IOP) elevation

Acute intraocular pressure (IOP) elevation was achieved by vitreous chamber cannulation in a randomly chosen eye. Studies in our laboratory have previously shown that anterior and vitreal chamber pressures have close agreement in our implementation (He et al. 2012). As previously described (He et al. 2012), cannulation was produced using a 27 G needle (Fig.1C) connected to a fluid reservoir (60 mL syringe) containing normal saline solution (Baxter International Inc. Toongabbie, NSW, Australia) via polyethelyne tubing (0.8 mm outer and 0.4 mm inner diameter, Unomedical, Sydney, NSW, Australia). Following cannulation, the desired IOP was achieved by placing a saline reservoir at a height precalibrated, using a mercury manometer (Livingstone, Sydney, NSW, Australia).

### Intracranial pressure (ICP) elevation

Intracranial pressure was manipulated via a custom-made dual-cannula placed into the lateral ventricle on the side ipsilateral to the eye cannula. In preparation for intracerebroventricular cannulation the rat was placed on a stereotaxic platform (Model 900, David Kopf Instruments, Los Angeles, CA) and the skin was excised (2 cm by 2 cm) and connective tissue around the calvarial area was removed by blunt dissection exposing the coronal sutures.

Using a dental burr (0.6 mm diameter) attached to a drill (Model 300, Dremel®, Robert Bosch Tool Corporation, Racine, WI), a hole was drilled through the skull at co-ordinates of 1.5 mm caudal to bregma and 2 mm lateral to midline. The needle was inserted to a depth of 3.5 mm which was located inside the lateral ventricle (Paxinos and Waston 2007) as evidenced by the stable ICP measurement during experimentation (Fig. S1). To ensure accurate ICP control over a prolonged period of time, a dual-lumen needle cannula was developed, which simultaneously infuses saline via a syringe pump (Model 22, Harvard Apparatus, Holliston, MA) and measures pressure in the same lateral ventricle. A 23G needle (0.6 mm diameter × 19 mm length, Becton Dickinson, Franklin, WI) was customized for the outer coat by manual filing to shorten the bevel (1 mm) and length of the needle (13 mm) (Fig.1B). This served as the measurement cannula and was bonded using cyanoacrylate (RS®, Sydney, NSW, Australia) to polyethelyne tubing (1.27 mm outer and 0.97 mm inner diameter, Unomedical). A 30G needle (0.3 mm diameter × 13 mm length, TERUMO®, Elkton, MD) formed the infusion cannula and was inserted into the outer 23G needle through the tubing to form the infusion cannula. This infusion cannula was connected to a smaller diameter polyethelyne tubing (0.8 mm outer and 0.4 mm inner diameter, Unomedical). The bevel of the inner needle was positioned perpendicular to the outer needle bevel, and the tips of two needles were aligned to a common level. To avoid leakage, cyanoacrylate was applied to seal all joints. A sleeve of polyethylene tubing was added around the outside of the 23G measurement needle to leave an exposed length of 3.5 mm from the tip of the needle (Fig.1B, cuff). Infusion pressure and ICP were continuously measured via two precalibrated pressure transducers (Fig.1A, Transpac, Abbott Critical Care System, Iligo, IRE) connected to a Powerlab system (Bridge Amp ML 110, Amplifier ML 785, Powerlab/8SP, ADInstruments, Colorado Springs, Colorado) and recorded on Lab Chart™ (Lab Chart 7, ADInstruments).

### Structural assessment: optical coherence tomography

Spectral domain optical coherence tomography (OCT) was utilized to investigate the deformation of the optic nerve head (ONH) and thickness changes in the retina (Image-Guided 830 nm OCT, Phoenix Research Laboratories, Pleasanton, CA). A line pattern was used to rapidly scan (1024-A-scans per b-scan, 10 repeats; axial resolution 4 *μ*m, lateral resolution 8 *μ*m) a selected cross section of the retina centered on the optic nerve, avoiding the larger vessels (see Fig. 3 right panels). In all cases, the line scan was orientated along the horizontal meridian. In one case, the intravitreal cannula obscured the view along the horizontal meridian and thus the scan was orientated at 45 degrees to lie between the major vessels.

It was observed that when ICP was elevated to levels above 15 mmHg, animal involuntary movements increased. Thus, the scan location and orientation were continuously monitored and every attempt was made to scan the same retinal location. Furthermore, to decrease random movement noise, a total of 10 images were taken at each IOP step. These were averaged during post hoc analysis using Fiji image processing software (Schindelin et al. 2012).

**Figure 2:**
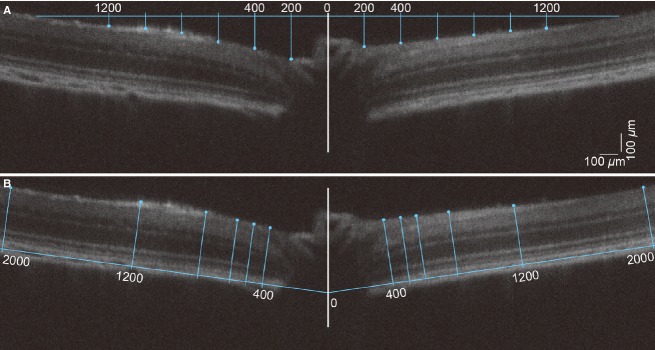
OCT analysis of surface position and retinal thickness. (A) Surface position was quantified relative to a horizontal reference line of 3200 *μ*m in length set to just touch the retinal surface at its extremities and centered on the optic nerve (horizontal blue line centered on the optic nerve). Measurements of retinal surface position were taken every 200 *μ*m (blue drop lines) from the midline (white line). (B) Retinal thickness was measured to the anterior surface of the retinal nerve fibre layer (blue dots) out to 2000 *μ*m either side of the center of the optic nerve perpendicular to a reference line drawn along Bruch’s membrane.

Figure2 shows the analysis approach used to measure surface position (Fig.2A) and retinal thickness (Fig.2B). Following averaging (10 B-scans), if required the OCT image (4000 × 500 *μ*m) was rotated so that the edges of the retinal plane lay at 180° and the image was centered on the optic nerve. To return surface position (Fig.2A), a 3200 *μ*m reference line (horizontal blue line) was drawn perpendicular to the vertical midline of the nerve (white-line). Every 200 *μ*m from midline, the distance from the horizontal reference line to the retinal surface was measured (Fig.2A). The 200 *μ*m surface measurement provides an assay of optic nerve head surface displacement as this area is within Bruch’s membrane opening (498.5 ± 2.5 *μ*m). In accordance with a previous study in rats (Fortune et al. 2011), the 400 and 1200 *μ*m measurements are referred to as central retina and peripheral retina, respectively.

To quantify retinal thickness (Fig.2B) reference lines along Bruch’s membrane were drawn (blue). At specified distances from the midline (every 200 *μ*m in central retinal area, but relatively sparse in the peripheral area) retinal thickness was measured as the distance perpendicular from the Bruch’s membrane reference line to the anterior surface of the retinal nerve fibre layer (blue dots). Note that the retinal surface position (Fig.2A) and retinal thickness (Fig.2B) measurement locations differ slightly for a nominal distance from the midpoint due to the angular orientations of the reference lines. For example, 400 *μ*m in Fig.2A does not correspond to the exact same retinal location as 400 *μ*m in Fig.2B.

### Functional assessment: electroretinography

Retinal function was assessed using the full-field electroretinogram. As described previously (Bui and Fortune 2004), custom-made silver-chloride active and reference electrodes were placed on the central cornea and sclera (ring shaped), respectively. These silver electrodes (99.9%, A&E Metal Merchants, Sydney, NSW, Australia) were connected to platinum leads (F-E-30, Grass Telefactor, West Warwick, RI). A stainless steel ground electrode (F-E2-30, Grass Telefactor, West Warwick, RI) was inserted subcutaneously into the tail.

Light stimulation was driven by an array of 8 white light LEDs (8 Watt Luxeon LED, Philips® Lumileds Lighting Company, San Jose, CA) and a dim LED (0.1 Watt Luxeon LED, Philips® Lumileds Lighting Company). The stimulus and signal capture were triggered simultaneously with Scope™ software (Powerlab ADInstruments). Data was collected with filter settings of 0.3–1000 Hz (−3 dB) with an amplification of (×1000) via pre-amplifiers (P511 AC Amplifier, Grass Telefactor). Signals were digitized (ML785 Powerlab 8SP, ADInstruments) and saved for post hoc processing. ERG waveforms were acquired with a 1 kHz sampling rate over a 640 msec recording window (2560 points), including a 10 msec prestimulus baseline.

Given the short interval between subsequent IOP steps (3 min) two stimulus energies were assessed to return measures of ganglion/amacrine cell (STR) and bipolar cell integrity (b-wave) (Stockton and Slaughter 1989; Kofuji et al. 2000; Bui and Fortune 2004). More specifically, the ganglion and amacrine cell STR was measured as the average of 20 flashes at a luminous exposure of −5.25 log cd s/m^2^ with a 2 sec inter-stimulus interval. This was followed immediately by a single moderate flash of −2.48 log cd s/m^2^, which is dominated by the rod bipolar cell response (He et al. 2006, 2008; Nguyen et al. 2008). Thus, ERG recordings took 45 sec to conduct, which allowed over 2 min for recovery before the next set of ERG measurements. This has previously been shown to be sufficient time for dark adaptation from these light energies (He et al. 2006, 2008), thereby ensuring that any attenuation of ERG response does not reflect light adaptation. Waveforms to dim flashes were analyzed by measuring the amplitude from the peak of the positive STR component to the trough of the negative STR. Moderate flashes were analyzed from baseline to the b-wave peak.

### Statistical analysis

All experimental data underwent normality (Kolmogorov–Smirnov test) and homogeneity of variance assessment (Bartlett’s test & Levene’s test). General linear model analysis (Minitab 16, Minitab Pty Ltd, Sydney, NSW, Australia) was employed as it is more robust to nonhomogeneous variance than ANOVA (Keppel 1991; Cnaan et al. 1997). Post hoc analysis was conducted, using Dunnett’s multiple comparison (within groups, IOP effect) and Tukey’s multiple comparison (across groups, ICP and ICP/IOP effect). Group data are given as mean ± SEM. An *F*-test was used to compare whether a two-line equation returned a significantly better description of the data compared with a simple linear function. Due to the nonparametric distribution of the data plotted against ONPG, the mean and 95% CI of the model parameters were returned by bootstrapping the group data (Efron and Tibshirani 1993). A Deming regression and Spearman correlation were adopted to determine associations between parameters.

## Results

### Effect of optic nerve pressure gradient modification on retinal structure

**Figure 3:**
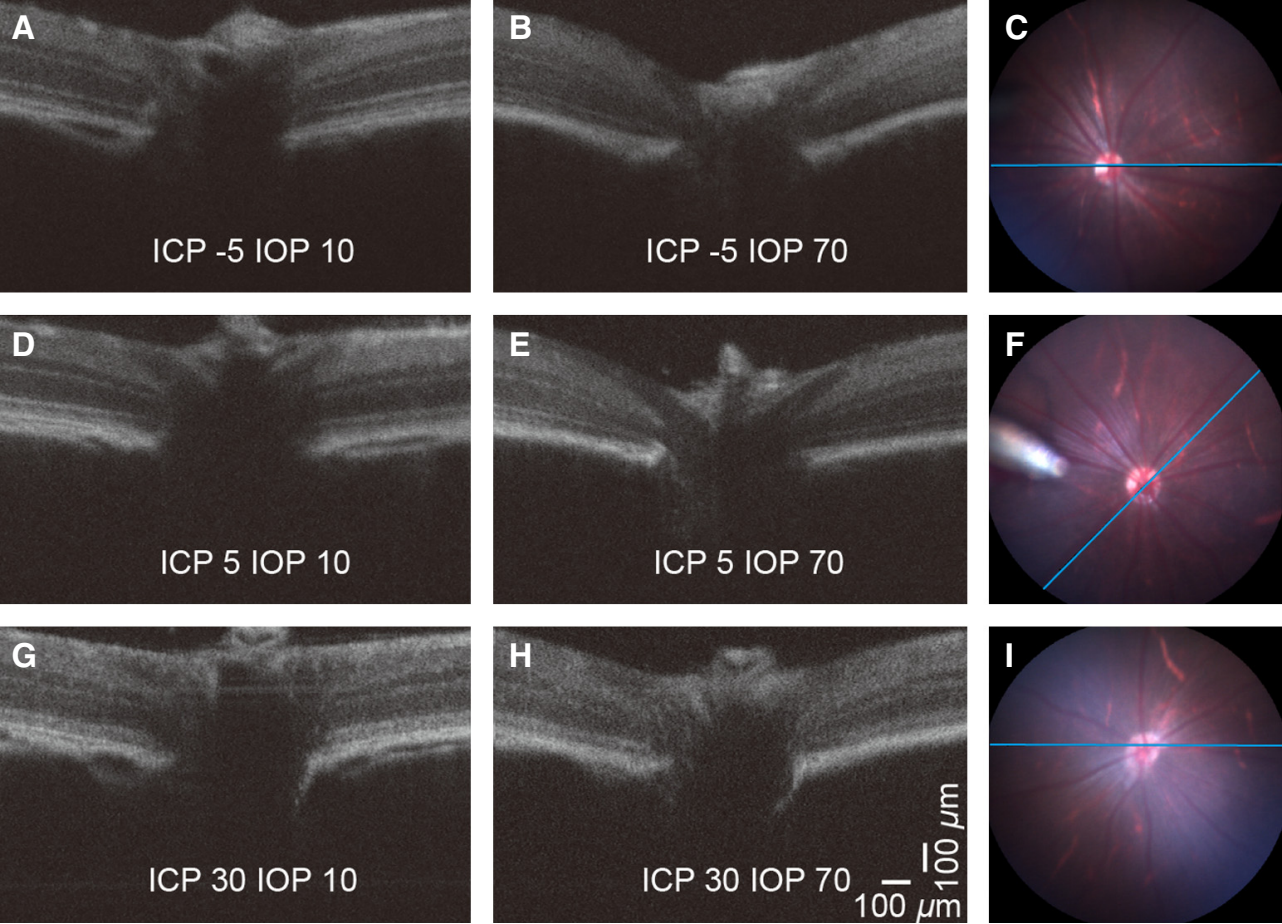
Representative OCT images showing the effect of IOP and ICP modification on the retina and ONH. Images in each row were acquired from the same rat, and the three rows indicate low (A, B; −5 mmHg) normal (D, E; 5 mmHg) and high (G, H; 30 mmHg) ICP levels. Panels on the right (C, F, I) show the corresponding full field fundus images. The blue reference line denotes the location of the OCT scan. In panel (F) the tip of the intravitreal cannula can be visualized at 9 o’clock. In this case the scan was taken at 45 degrees to the horizontal. In all other cases the reference was within 10 degrees of horizontal. The left and middle columns compare the effect of normal (A, D, G; 10 mmHg) and elevated IOP (B, E, H; 70 mmHg) on the structure of the peripapillary tissue and the optic nerve head. Note the backward bowing and remarkable retinal compression at IOP 70 mmHg (E) which is partially prevented by raising ICP (H).

Figure3 shows representative OCT images serially recorded at baseline (first column) and elevated IOP (second column = 70 mmHg) in three separate animals that had ICP maintained at low (−5 mmHg), normal (5 mmHg) and high (30 mmHg) levels. In the rat with a low and normal ICP (Fig.3A–C and D–F), it is clear that the surface of the optic nerve and the retina close to the ONH are displaced posteriorly with IOP elevation. In the animal with low ICP, 70 mmHg IOP appears to compress the major vessels that are found in the middle of the optic nerve. In the animal with very high ICP, there was only a small posterior deformation even when IOP was raised to 70 mmHg (Fig.3G–I).

**Figure 4:**
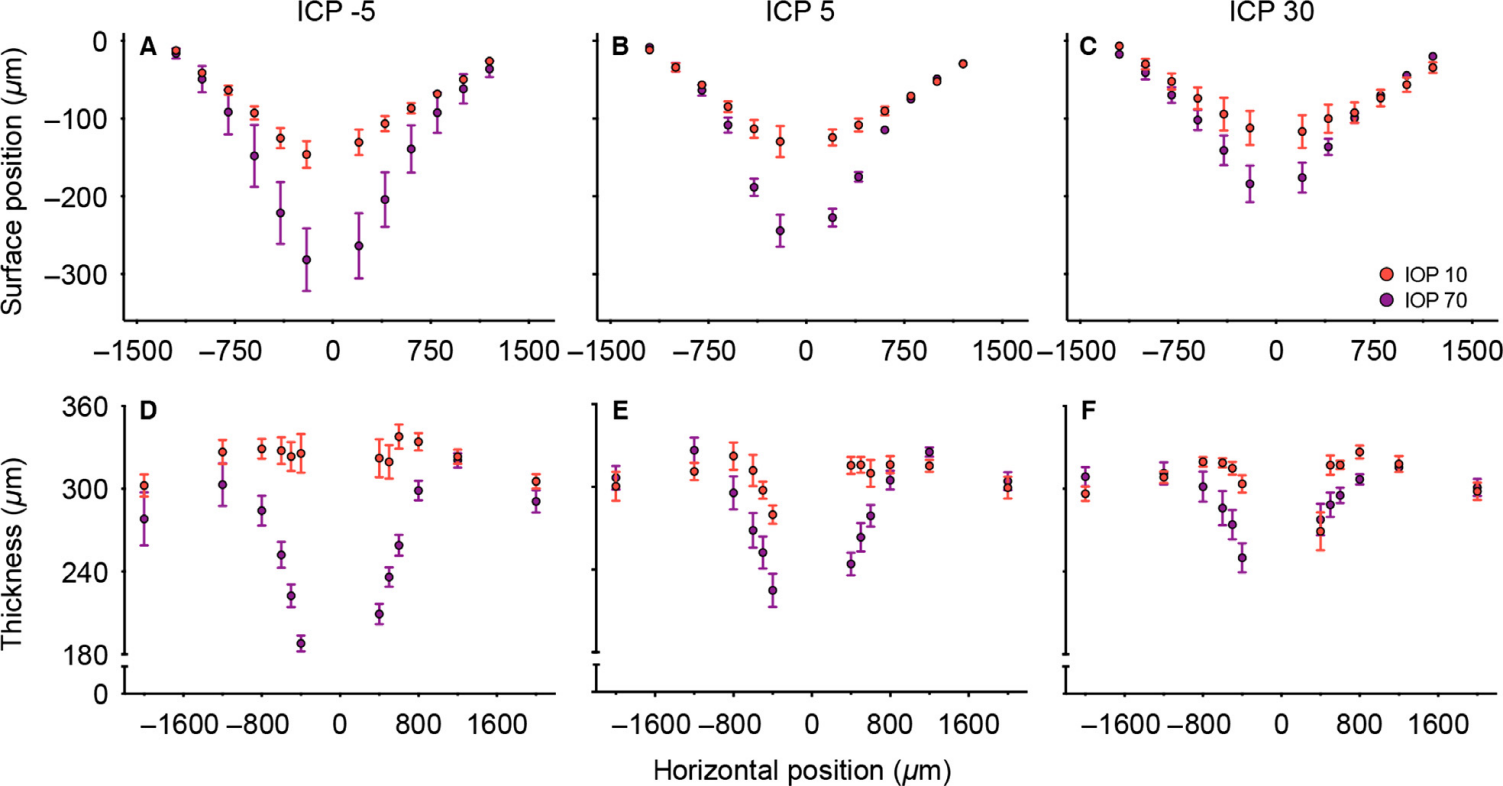
Group average effect of IOP and ICP on surface position and retinal thickness. Data are shown as mean (±SEM) for three selected ICP levels (low −5, normal 5 and high 30 mmHg) and two selected IOP values (normal 10 and high 70 mmHg). (*n* = 6–7). The complete range of IOP levels is illustrated in Fig. S3. For each ICP level (mmHg) the IOP is denoted by 10 = red, 70 = purple. Surface position is shown in panels (A–C) whereas panels (D–F) show retinal thickness.

For clarity, group data for three selected ICP levels (low, normal, high) and two key IOP levels (normal, high) are shown in Figure4. The complete range of IOP levels is shown in Figure S3. The surface position of the rat peripapillary retina and optic nerve show a “V-shape”. Surface position is progressively lower with IOP elevation for regions within ∼1200 *μ*m from the midline (Fig. S3). This effect was similar for both temporal and nasal sides of the optic nerve. When comparing different ICP levels, it is apparent that IOP-induced surface deformation is more marked with low (Fig.4A) and less so with high ICP (Fig.4C).

Images were also analyzed for retinal thickness and the data are shown in Figure4D–F. In Long-Evans rats, the retinal thickness is about 300 *μ*m at approximately 750 *μ*m from the center of the optic nerve. The retina became progressively thinner with IOP elevation (Fig. S3). This effect was more prominent closer to the optic nerve. In comparison to the normal ICP group (Fig.4E), there was more retinal compression in animals with lower ICP (Fig.4D), whereas there was less retinal compression in animals with higher ICP (Fig.4F). This ICP difference appears more marked at higher IOP levels (70 mmHg, purple circle) than at normal IOP (10 mmHg, red circles). When we compare surface position and retinal thickness at baseline IOP (10 mmHg) across ICP levels, there is a trend for higher surface position and a thicker retina with increasing ICP. However, this effect was not significant as shown in Figure S2.

**Figure 5:**
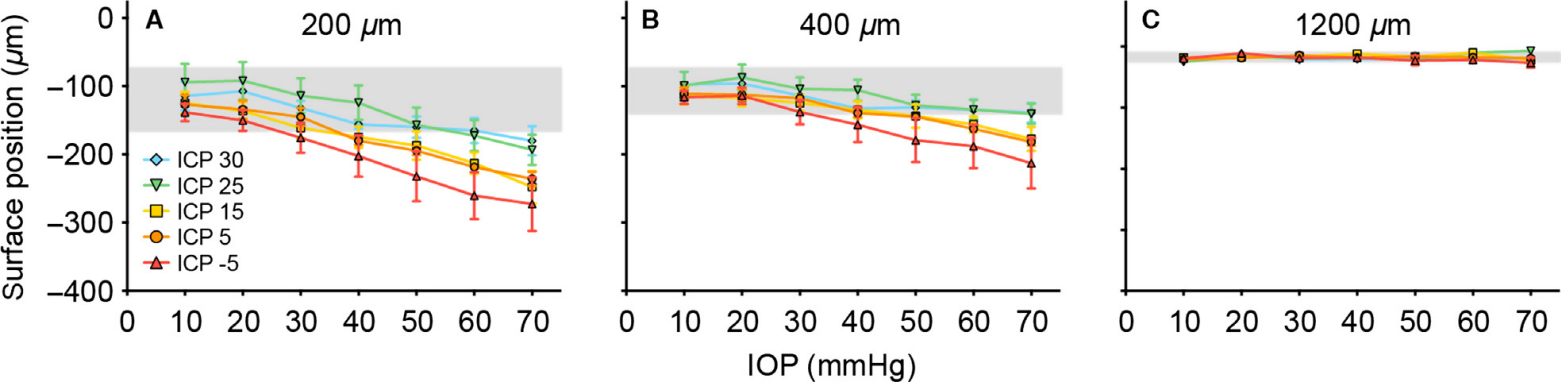
Effect of ICP on IOP-induced changes in surface deformation. Panels (A–C) (*n* = 6–7) show raw surface deformation at (A) the optic nerve head 200 *μ*m from midline. (B) the central retinal 400 *μ*m from midline and (C) the peripheral retina 1200 *μ*m from the midline. (mean ± SEM). Blue diamond: 30 mmHg; green down triangle: 25 mmHg; yellow square: 15 mmHg; orange circle: 5 mmHg; red up triangle: −5 mmHg. Gray area: 95% confidence interval of the baseline (IOP = 10 mmHg).

**Figure 6:**
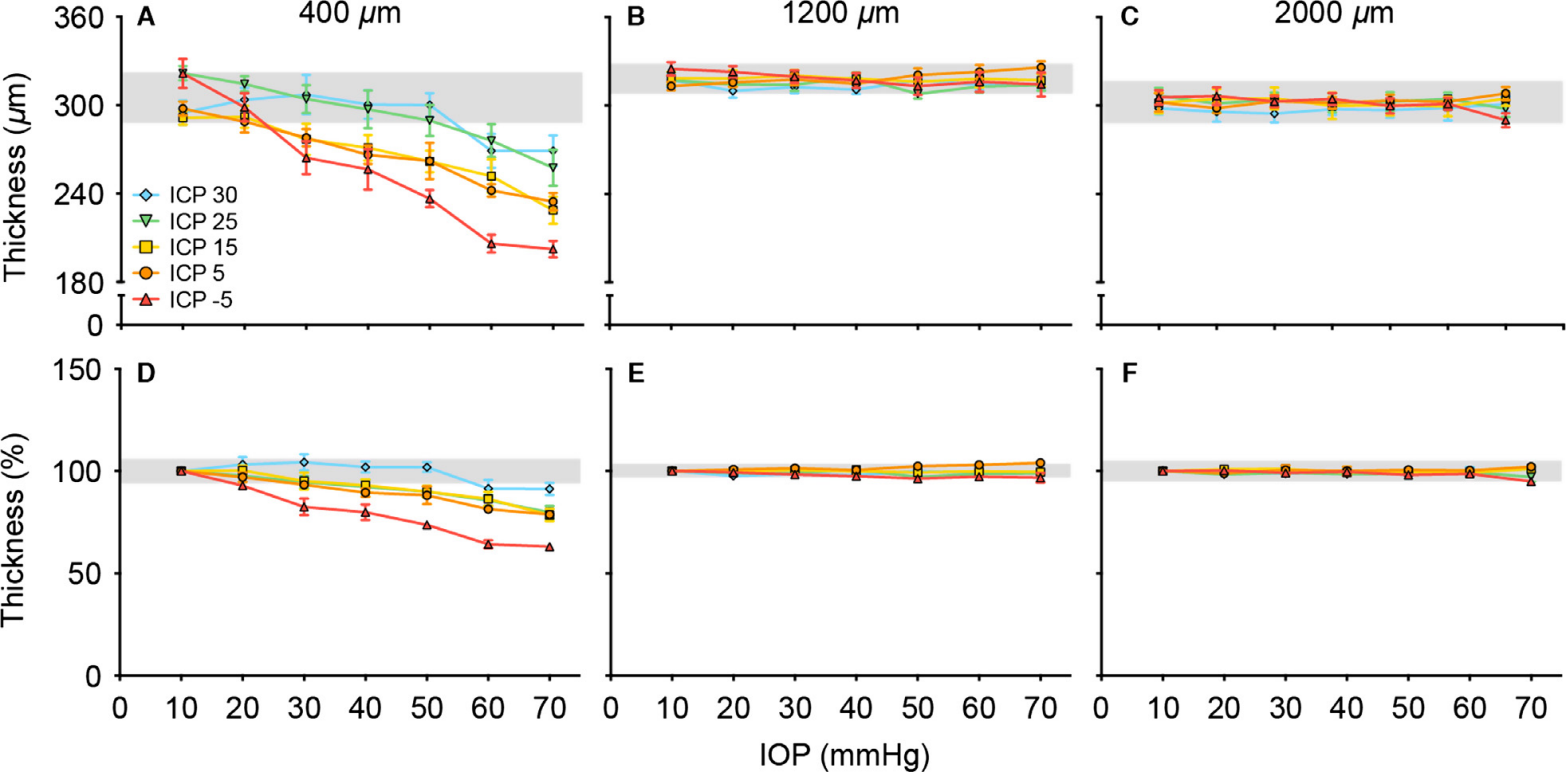
Effect of ICP on IOP-induced changes in retinal thickness. (A) Retinal thickness at 400 *μ*m, (B) 1200 *μ*m and (C) 2000 *μ*m from the midline. (D–E) show retinal thickness normalized to baseline IOP for the same three locations (*n* = 6–7 in each group, mean ± SEM). Retinal thickness was averaged from both temporal and nasal sides of the optic nerve for each eye.

A subset of the group data is plotted in Figures5 and 6 to highlight how ICP modifies the effect of IOP elevation at three key locations. As no significant change to retinal thickness was found at baseline IOP (see Fig. S2), data are also expressed normalized to IOP of 10 mmHg (Fig.6D–F). The “percentage change” in surface position is dependent on the arbitrary horizontal reference line (Fig.2A), so normalization was not used for this parameter. Figure5A shows increasing optic nerve head surface deformation with increasing IOP (*P* < 0.001, illustrated by the downward trend of all the data) and with lower ICP (indicated by warmer colors lying below cooler colors). There was no interaction between ICP and IOP (*P* = 1.000). Similarly in the peripapillary retina (Fig.5B), both IOP and ICP affected retinal surface position (IOP: *P* < 0.001; ICP: *P* < 0.001) and no interaction effect was observed (*P* = 0.998). In the peripheral retina (1200 *μ*m, Fig.5C), there was no significant change in surface position with IOP elevation or change in ICP level (IOP: *P* = 0.166; ICP: *P* = 0.192, interaction: *P* = 0.064).

Figure6 shows retinal thickness at three key retinal locations. Retinal thickness at a central location (400 *μ*m, Fig.6A) showed a decrease with IOP elevation in all ICP groups. There was significantly greater thinning in animals with ICP of −5 mmHg (red), and less thinning in animals with higher ICP (cooler colors) as shown in Figure6A and D, respectively. That ICP modified the degree of IOP-induced retinal thinning was evidenced by a significant interaction effect between IOP and ICP (*P* = 0.003). At a more peripheral retinal location of 1200 *μ*m (Fig.6B, E), there was less retinal thinning but a significant ICP effect when normalized to baseline (Fig.6E, *P* < 0.001). At 2000 *μ*m, there was no retinal thickness change in response to change in IOP or ICP (Fig.6F*,* IOP: *P* = 0.995, ICP: *P* = 0.343, interaction: *P* = 0.998).

### Effect of optic nerve pressure gradient modification on retina function

**Figure 7:**
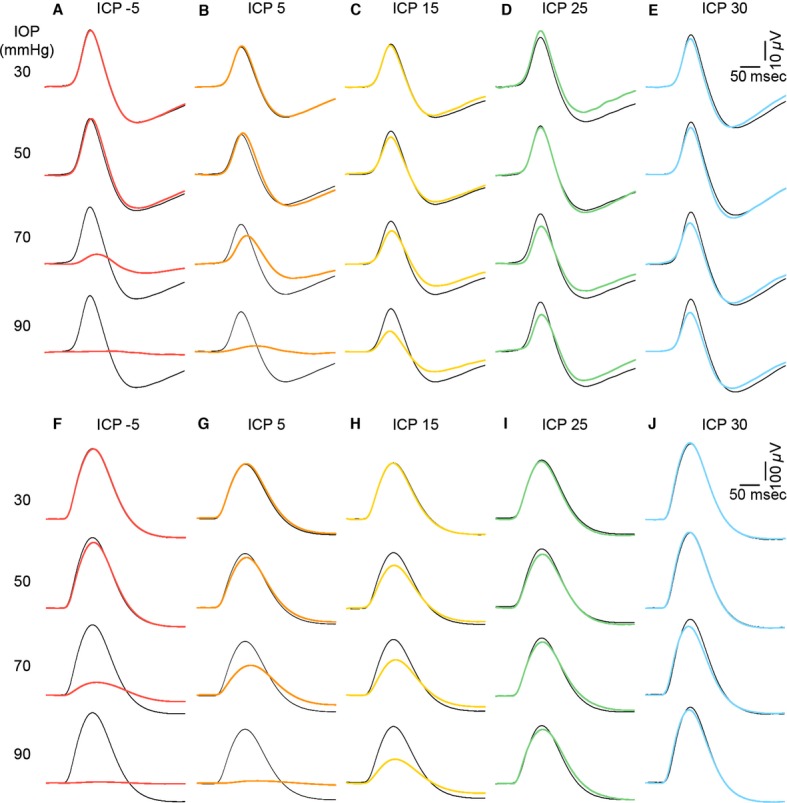
Effect of ICP modification on IOP-induced changes to electroretinogram waveforms. Group average ERG waveforms of panels (A–E), scotopic threshold responses indicating proximal retinal function. (F–I) b-wave representing ON-bipolar cell function. Black thin traces indicate waveforms measured at baseline (IOP = 10 mmHg) for each ICP group. Colored traces indicate ERG responses following IOP elevation. Warmer colors represent low ICP and cooler colors high ICP levels.

Figure7A–E show averaged STR waveforms from various ICP groups during IOP elevation. With normal ICP (5 mmHg, orange waveforms), the STR was unaltered for IOP levels of 50 mmHg or less. Reduction and delay in both positive and negative features of the STR are readily apparent at an IOP of 70 mmHg, when compared to baseline waveforms (IOP = 10 mmHg, black thin trace). Greater attenuation of the STR was observed when IOP was raised to 90 mmHg. That higher IOPs produced greater dysfunction was apparent in all ICP groups. Comparison between ICP levels demonstrates that in animals with low ICP (warmer colors), the STR was more susceptible to IOP elevation; the converse was true for animals with higher ICP (cooler colors). This difference in functional susceptibility was particularly visible at higher IOPs. At an IOP of 70 mmHg, there was a ∼90% attenuation of the STR in animals with an ICP of −5 mmHg, as compared with a ∼50% reduction in animals with normal ICP of 5 mmHg. However, in those groups with higher ICPs (15, 25 and 30 mmHg), there was little STR loss (<10%). Consistent with the changes seen in the STR, Figure7F–J show that low ICP makes the electroretinogram b-wave more sensitive to IOP elevation, whereas higher ICP levels make the b-wave less sensitive to IOP elevation.

**Figure 8:**
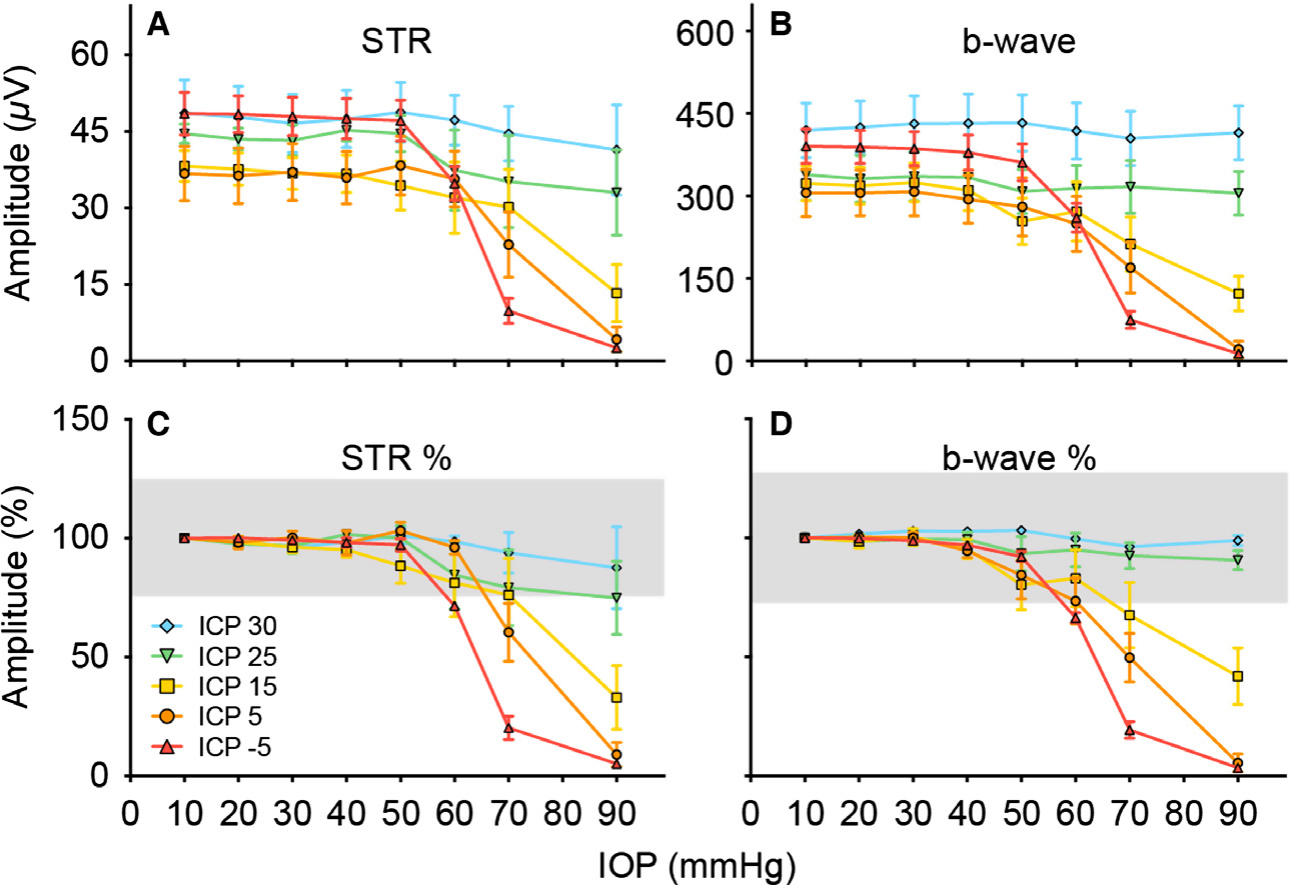
Group average effects of IOP and ICP on the electroretinogram. Mean amplitudes (±SEM, *n* = 5–9 in each group) of for the raw STR (A) and b-wave (B). These data were also expressed normalized to baseline (IOP = 10 mmHg) (C and D). The grey area indicates the 95% confidence interval of baseline amplitudes (IOP = 10 mmHg).

Figure8 summarizes the changes in STR (Fig.8A) and b-wave (Fig.8B) amplitude. As there was no significant difference between the baseline amplitude of the ERG components between the various ICP groups, data were normalized to the amplitude at IOP 10 mmHg as shown in Figure8C and D. This figure confirms that ERG amplitudes showed progressively greater attenuation with higher IOP elevation and that ICP level modifies the effect of IOP elevation on retinal function. More specifically, there was a significant interaction between IOP and ICP levels for both STR (*P* < 0.001) and b-wave (*P* < 0.001). This indicates that IOP and ICP effects are interdependent on each other.

**Figure 9:**
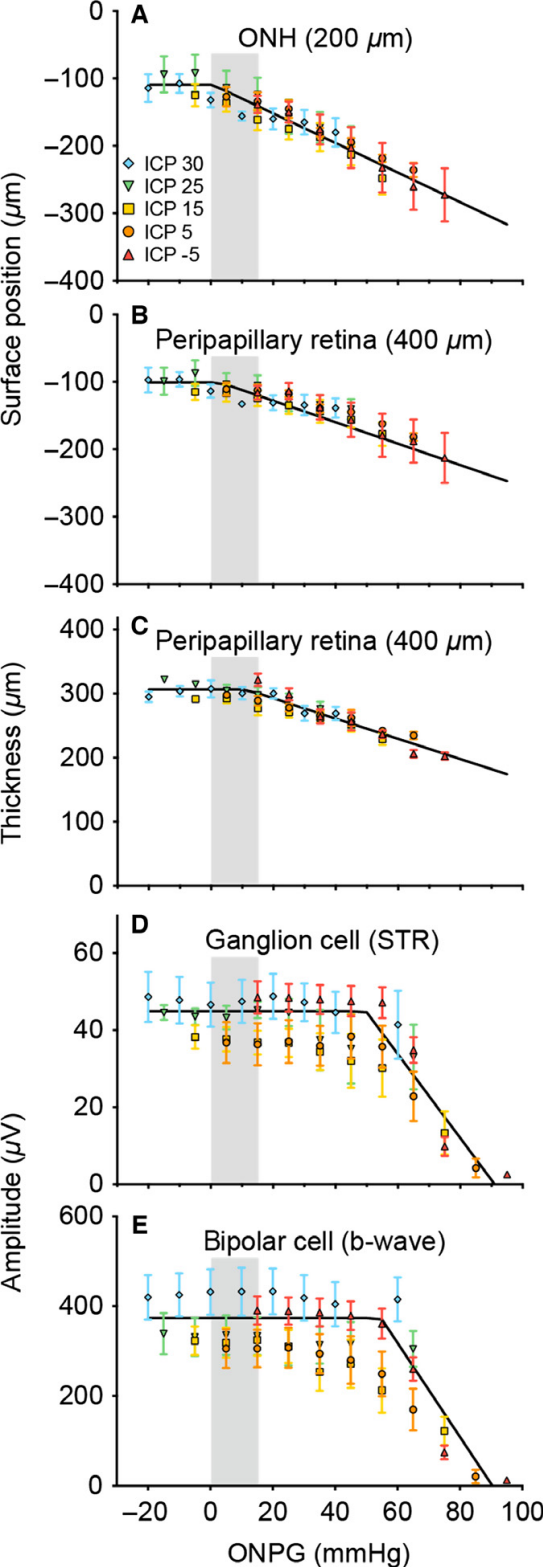
Effect of optic nerve pressure gradient modification on retinal structure and function. The effect that IOP and ICP have on structural and functional assays are compared by plotting parameters against optic nerve pressure gradient (ONPG = IOP − ICP). Data are described using a two-line function where the intercept represents the critical ONPG threshold where effects become apparent. (A–B) ONH and peripapillary retina surface position, respectively. ONPG threshold for ONH is −0.6 mmHg and peripapillary retina = 3.0 mmHg. (C) thickness of the peripapillary retina (ONPG threshold = 11.3 mmHg). (D) Normalized retinal ganglion cell-mediated (scotopic threshold response, STR) function (ONPG threshold = 49.7 mmHg). (E) bipolar cell-mediated (b-wave) function (ONPG threshold = 54.6 mmHg). Grey area indicates normal range of ONPG (0–15 mmHg).

To examine whether the effects of IOP and ICP modification simply reflect the ONPG (IOP - ICP), OCT and ERG parameters are re-plotted against ONPG in Figure9. Raw data are considered here and normalized data are shown in Figure S4. When plotted against ONPG, Figure9 and Figure S4 show that data points from the various ICP groups show a common trend for surface position, retinal thickness, and ERG parameters. It is apparent that in relation to increasing ONPG structural and functional parameters show a plateau before a decline. A two-line function provides statistically smaller residual errors compared with a simple linear regression for all parameters (*F*-test, *P* < 0.001).

The two-line function is useful as its intersection point provides an estimate of the critical threshold ONPG for change. Structural parameters (surface deformation and retinal thickness) appear to exhibit lower thresholds, and therefore greater susceptibility to ONPG elevation than functional parameters. More specifically, the threshold was –0.6 (95% confidence limits, −21.8 to 37.3) mmHg for ONH surface position, 3.0 (−24.4 to 50.2) mmHg for peripapillary surface position, and 11.3 (−1.7 to 22.4) mmHg for retinal thickness. The STR (49.7, 24.1–60.0 mmHg) and b-wave (54.6, 39.2–60.0 mmHg) both return threshold values that lie beyond the upper limit for all structural parameters (*P* < 0.05).

**Figure 10:**
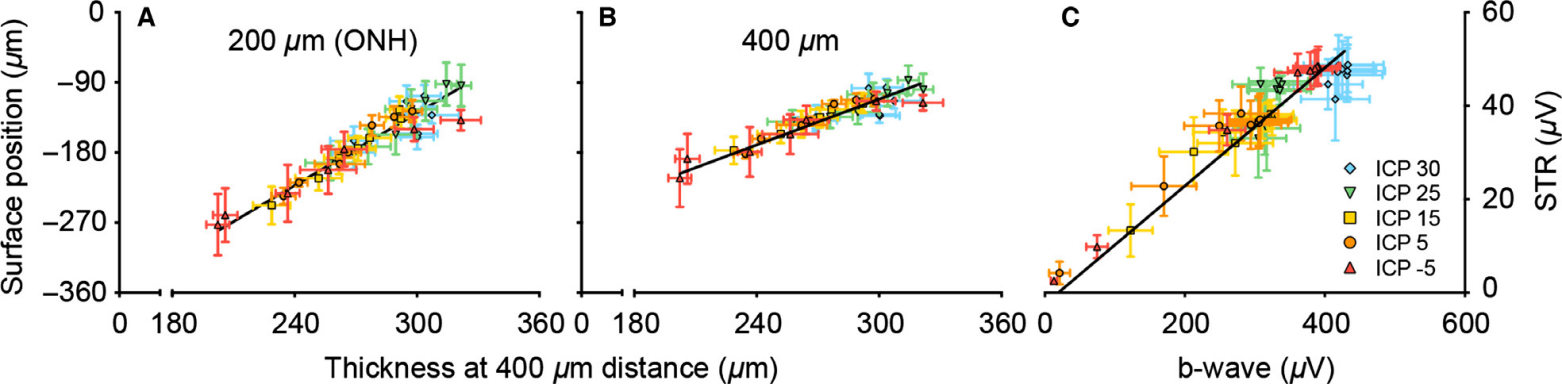
Association between structural parameters and between functional parameters. (A–B) Retinal thickness in the peripapillary area (400 *μ*m) correlated against surface deformation at the optic nerve head (A, 200 *μ*m) and peripapillary area (B, 400 *μ*m). (C) Bipolar cell function returned by b-wave amplitude correlated against retinal ganglion cell function (STR amplitude). Data expressed as mean amplitudes (±SEM). Lines are Deming regression (*P* < 0.001 for all panels).

Figures10 and 1 further explore the relationship between structural and functional outcomes, by correlating them against each other. Figure10A and B show that surface position and retinal thickness are strongly correlated with each other (Fig.10A, B; *r*_s_ = 0.92–0.93, *P* < 0.001). Similarly, Figure10C shows that ganglion cell function is strongly correlated with bipolar function (Fig.10C; *r*_s_ = 0.92, *P* < 0.001). In contrast, in Figure1 when any of the structural parameters (surface deformation and thickness) were correlated against either functional parameter (STR or b-wave), the relationships appear to be more curvilinear.

**Figure 11:**
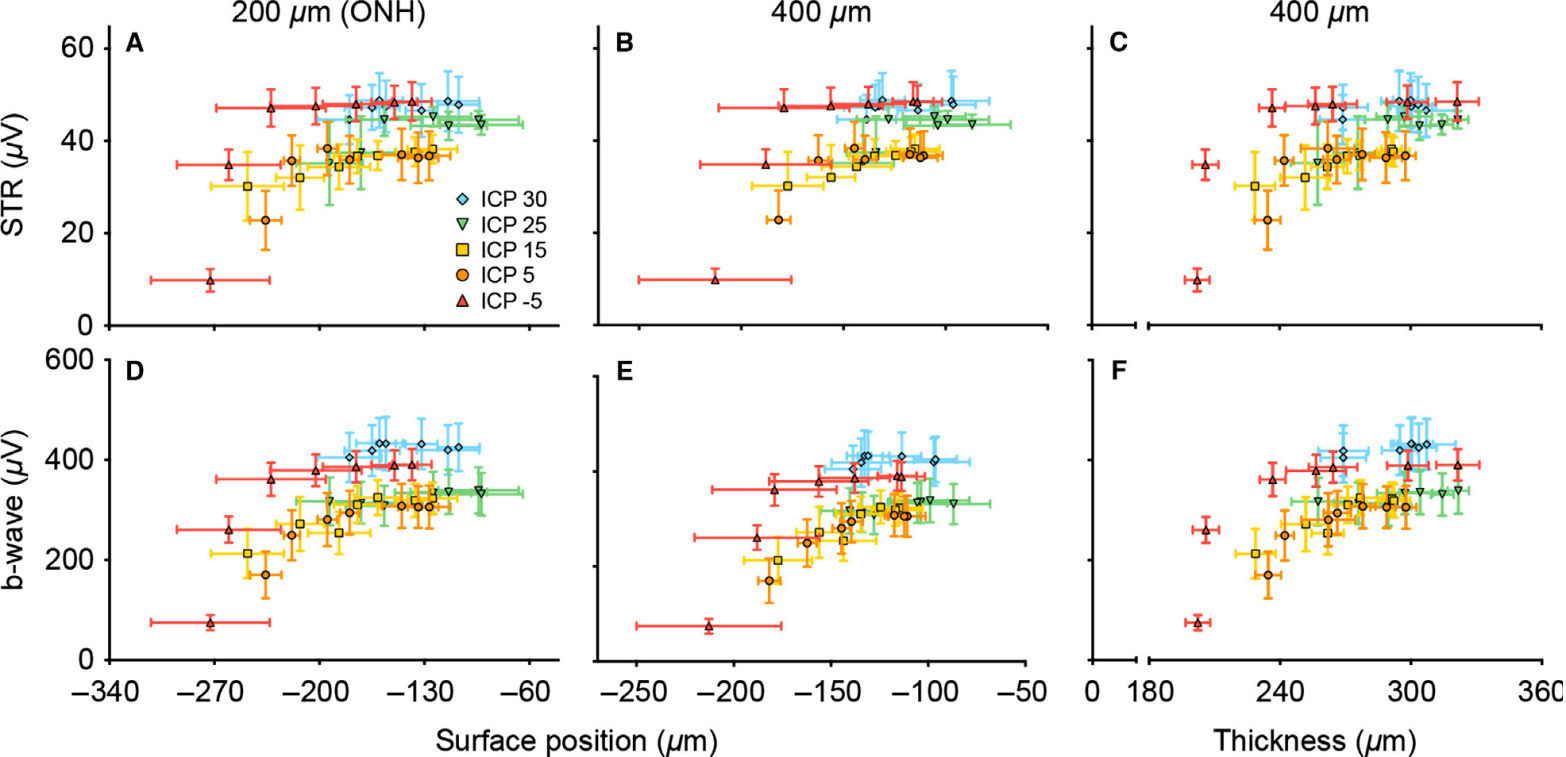
Structure-function relationships with ONPG modification. Structural parameters (on *x*-axis) of surface position and thickness are plotted against functional output in terms of ERG amplitude (on *y*-axis) of ganglion and bipolar cells. All structure-function relationships exhibit a curvilinear relationship. Data expressed as mean (±SEM).

## Discussion

### Effect of acute IOP and ICP modification on retinal structure

Posterior movement of the ONH surface and surrounding peripapillary retina during IOP elevation, is exacerbated by low ICP, and ameliorated by high ICP. Previous studies have shown that both acute IOP elevation (Heickell et al. 2001; Burgoyne et al. 2005) and laser-induced chronic IOP elevation in non-human primates (Yang et al. 2011) causes posterior displacement of optic nerve and peripapillary tissue. In rodents, Fortune et al. (2011) demonstrated posterior displacement of the ONH surface and outward bowing of peripapillary tissue (10° around the ONH) when IOP was raised to 50 mmHg. Consistent with previous studies (Fortune et al. 2009, 2011) we show that tissue deformation with IOP elevation was greatest at the ONH (200 *μ*m) and peripapillary retina (400 *μ*m, ∼10°) and progressively less with increased retinal eccentricity.

It is clinically established that chronically elevated ICP induces papilledema (Kupersmith et al. 2011). MRI studies in patients with idiopathic intracranial hypertension have shown that high ICP produces flattening of the posterior sclera and optic nerve protrusion (Alperin et al. 2013). Given this we would have expected increasing anterior displacement with increasing ICP at baseline IOP. Although a trend was evident (Fig.5A, IOP 10 mmHg and Fig. S2) this was not statistically significant. Furthermore, the glaucoma literature indicates that patients with low ICP coupled with normal IOP may exhibit glaucomatous changes (Berdahl et al. 2008b; Ren et al. 2010; Wang et al. 2012). Again there is a trend in the current study for greater ONH surface displacement in our low ICP group (−5 mmHg) at normal IOP (10 mmHg) however this did not reach significance (Fig. S2) which may reflect the acute nature of our experiment compared to the chronic changes reported in the literature. Indeed, when all IOP levels are taken into consideration there is a relatively anterior displacement of the ONH and peripapillary retina surface with increasing ICP (*P* < 0.001) suggesting we were simply underpowered to detect differences when ICP was changed at baseline IOP. What is clear is that high ICP groups (25 and 30 mmHg) showed significantly less peripapillary deformation compared to normal (5 mmHg) and low ICP (−5 mmHg) groups, even when IOP was 70 mmHg (Fig.5: post hoc analysis, *P* < 0.05).

Only two studies have investigated the effect that both ICP and IOP modification have on ONH structure with in vivo imaging. Morgan et al. (2002) considered the effect of acute pressure manipulation on the canine optic nerve. These authors found that ONPG was the major determinant of ONH surface position with ICP having a slightly greater effect than IOP. This is in accordance with our findings in the rodent. This study is qualitatively in agreement with 12 months of chronic ICP lowering in non-human primates, which produced posterior deformation of the ONH and peripapillary retina (Yang et al. 2014).

It is well documented that IOP elevation causes retinal thinning, especially in the retinal nerve fibre layer (Fortune et al. 2009, 2011; Strouthidis et al. 2011). In agreement with these studies we found that acute IOP elevation produced a dramatic reduction in peripapillary retinal thickness in low and normal ICP groups. This effect arises from tissue compression with acute increase in IOP (Fortune et al. 2011). Fortune et al. (2009) demonstrated that significant retinal compression occurs within 800 *μ*m from the center of the ONH in non-human primate. Here we observe a similar effect, that retinal compression was greater nearer the ONH of rats (Fig.6). As the ONH is the weakest part of a pressurized sphere more stress and strain is manifest here (Burgoyne et al. 2005; Downs et al. 2008), and thus greater tissue compression is seen nearer the optic nerve. The degree of peripapillary retinal compression and ONH surface displacement with high IOP is substantial, and may suggest that in addition to compression there is peripapillary tissue displacement toward the scleral canal.

We extend previous work and show that changing ICP can modify the degree of retinal compression induced by acute IOP elevation (Fig.6). There is a 37% reduction in retinal thickness when IOP was elevated to 70 mmHg in animals with low ICP (−5 mmHg). In contrast, with high ICP (30 mmHg), IOP elevation to 70 mmHg produces only ∼11% retinal thinning. Thus ICP modifies the manner in which the ONH and peripapillary retina responds to IOP elevation. This does not occur at more peripheral retinal locations (>1200 *μ*m, Fig. S3), where neither IOP nor ICP changes exhibited an obvious effect on thickness. As ICP affects a restricted area of the posterior pole via the subarachnoid space, it is not surprising that the peripheral retina (1200 *μ*m) is not affected by ICP modification (Figs.4, 5). This is in agreement with the idea that increased ICP will add an inward mechanical force on the ONH and peripapillary sclera.

The manner in which ICP modifies surface position is similar to its effect on retinal thickness. Indeed change in ONH surface position and change in peripapillary retinal thickness show a strong linear relationship (Fig.10A and B). This linear relationship argues for peripapillary tissue displacement in addition to compression. If IOP elevation only produced compression of the optic nerve and peripapillary tissue then retinal thinning and surface position should depend only on IOP, and not ONPG as seen here (Fig.9). One way in which high ICP can prevent the IOP-induced changes is to minimise expansion of the optic nerve sheath posterior to the sclera, and thus help prevent peripapillary tissue displacement. Further studies utilising fiducial marks would help to define the exact nature of the conformational changes seen here.

### Effects of acute IOP and ICP modification on retinal function

It is widely accepted that acute IOP elevation induces retinal dysfunction, with the inner retina being particularly susceptible (Bui et al. 2005; He et al. 2006, 2008). Consistent with previous studies (He et al. 2012) severe reduction in both the STR and b-wave occur at an IOP of 70 mmHg and all responses are abolished at an IOP of 90 mmHg (Fig.8).

Previous studies have also demonstrated that the STR is the more sensitive component to acute IOP elevation (Bui and Fortune 2004; Bui et al. 2005; He et al. 2006). The current data provide some support for this. Figure8C shows that for an IOP of 90 mmHg the STR in animals with ICP of 25 and 30 mmHg was 74.9 ± 15.4% and 87.6 ± 17.2% of baseline. For the same IOP level, the b-wave was less affected in groups of rats with ICP of 25 mmHg (90.7 ± 4.0% of baseline) and 30 mmHg (98.9 ± 2.2% of baseline, Fig.8D). At low and normal ICP levels, there was little difference in the sensitivity of the STR and b-wave to IOP elevation. When STR and b-wave amplitude are plotted against ONPG, the ganglion cell response was slightly more sensitive to ONPG modification (Fig.9: intersection of 2-line model, 49.7 mmHg) than was the bipolar cell response (54.6 mmHg) however this difference was not statistically significant.

Modification of ICP dramatically alters the magnitude of retinal dysfunction induced by IOP elevation. With higher ICP levels, there was protection for retinal function against IOP elevation (Fig.8C and D). An ICP of 30 mmHg could completely ameliorate the total loss of retinal function induced by an IOP of 90 mmHg. To our knowledge, this is the first study to demonstrate that ICP modification has such marked effects on retinal function. The IOP and ICP levels used in this study are deliberately extreme as a proof of concept, which enables a wide range of ONPG to be evaluated and comparison of structural and functional susceptibility to be more comprehensively considered. Indeed, human diseases such as glaucoma or intracranial hypertension typically exhibit more subtle pressure changes over a longer time period. Nevertheless, the current study clearly highlights the importance of ICP to retinal physiology. Our data suggest that a difference in ICP of 10 mmHg can substantially change the functional response to IOP elevation. Morgan et al. (2002) have shown that the volume of the canine optic nerve can be modified by a change in ICP of as little as 2–5 mmHg. Whether such small differences in ICP over a protracted time course increases the risk of optic nerve injury requires investigation. Nevertheless, clinical differences in ICP between control subjects and those with glaucoma is in the order of 3–4 mmHg (Berdahl et al. 2008b; Ren et al. 2010).

### The effect of optic nerve pressure gradient on structure and function

The relationship between retinal structure and function and ONPG is explored in Figure9. It can be seen that ONPG is the major determinant of both biomechanical and functional changes in the rat retina as the various ICP groups overlap. Structural and functional parameters exhibit two distinct phases in response to ONPG modification. The intersection point of the two-line function provides an index of the threshold ONPG at which structural and functional parameters decline. Structural parameters are more sensitive to ONPG modification (Fig.9A–C) with the transition point occurring between −0.6 and 11.3 mmHg. This almost falls within the normal ONPG range for anesthetized rodents (∼0–15 mmHg) assuming basal IOP = 10–15 mmHg (Cohan and Bohr 2001) and basal ICP = 0–10 mmHg (Kalmar et al. 2009; Silasi et al. 2009; Chowdhury et al. 2013). This would suggest that small increases in ONPG would induce ONH surface deformation and peripapillary retinal compression. Such a finding is in agreement with the literature, where pressure changes of 2–5 mmHg can cause displacement of lamina cribrosa position in a canine model (Morgan et al. 2002). In contrast, the functional parameters are less sensitive to ONPG elevation, with thresholds of 49.7 (STR) and 54.6 mmHg (b-wave). Thus, retinal function appears to be protected against a certain amount of ONH surface deformation and peripapillary retinal compression. More specifically, by extrapolating the normalized functional ONPG threshold (Fig. S4, average between ganglion and bipolar cell response) of 51.3 mmHg onto retinal thickness, it can be seen that a 20.2% retinal compression can occur before function declines in the rat. Further studies are required to elucidate the compensatory mechanism by which neural function is preserved to some extent despite the tissue compression. It should also be noted that the changes to structural parameters including deformation and thickness may be reflective of not only mechanical tissue alterations but also simultaneous vascular changes that are altered with both ICP and IOP manipulation. We have previously shown, that blood flow and oxygen extraction closely follow functional decline with IOP elevation (He et al. 2013; Lim et al. 2014). Further studies are required to examine the role that vascular factors play in ONPG modification.

Expressing structural and functional changes against ONPG (Fig.9) helps to explain why a small increase in ICP can ameliorate changes seen with a large IOP elevation. For example, in Figure8D an ICP of 30 mmHg (blue diamond) is sufficient to completely ameliorate the b-wave loss when IOP was elevated to 90 mmHg in animals with normal ICP (5 mmHg, orange circles). This also occurs to a lesser extent with structure where an ICP of 30 mmHg (Fig.5A) ameliorates the subtle optic nerve head displacement induced by an IOP of 40 mmHg. This latter finding is consistent with Morgan et al. (2002) who showed that the surface of the canine optic nerve was slightly more sensitive to changes in ICP than IOP. At first glance, one may be led to believe that this means ICP exerts a stronger forward force on the nerve than an equivalent IOP does on backward force, a theory disproved in Figure9A as all ICP groups lie along the same ONPG curve. In fact, the observation that small changes in ICP can ameliorate larger changes in IOP can be attributed to the fact that there is a range of ONPG’s over which normal structure and function is maintained creating an ONPG threshold or “buffer-zone”. For example, if the OCT/ERG relationships with ONPG in Figure9 were simply a linear equation, then an equivalent change in IOP or ICP would alter OCT/ERG equally. However, because there is a “buffer-zone” there is a range of ONPGs over which no structure or function changes occur. Using the b-wave as an example (Fig.9E), in this manner not only does a normal ONPG of 0–15 mmHg (take equivalent IOP = 10 and ICP = 10 for simplicity, which returns ONPG = 0 mmHg) produce no functional loss but an elevated ONPG of 60 mmHg also produces no change, so a very high IOP of 90 mmHg can be completely ameliorated by a lesser change in ICP to only 30 mmHg. Thus, this two-line relationship gives the impression that ICP exerts a stronger force than IOP. As the ONPG buffer zone or threshold is much larger for STR and b-wave amplitude (49.7–54.6 mmHg) than it is for the structural parameters (−0.6 to 11.3 mmHg), this disparity between ICP and IOP effect is particularly exacerbated in the functional outcomes.

Figure10 shows that structural parameters are linearly related to each other (Fig.10A and B), as are functional parameters (Fig.10C). When structural parameters are correlated against functional parameters, a more complex curvilinear relationship is found. Further studies are required to consider whether chronic pressure modification also produces a curvilinear relationship between structure and function. It is of interest that correlations between visual field sensitivity or ERG with ganglion cell loss can exhibit linear or curvilinear relationships depending on the units used and the eccentricity of deficits (Swanson et al. 2004; Drasdo et al. 2007; Malik et al. 2012).

In summary, the influence that ICP has on the eye, and particularly the role that it may play in glaucoma is an emerging area of interest. Here we examine for the first time, the effect that acute ICP and IOP modification have on both structure and function of the retina and optic nerve head. In accordance with the literature, minor changes to ICP level exert more substantial effects on optic nerve structure than equivalent changes in IOP. This disparity between the effect of ICP and IOP is even more apparent when we assess their effect on retinal function. This can be explained by elevated ICP shifting the IOP curve toward the plateau that precedes the critical threshold for optic nerve structural change and functional loss (Fig.9). Structural measures such as surface displacement and tissue compression are more sensitive to ONPG modification than are electrophysiological measures of retinal function. Further studies are required to examine the relationships between structure and function in a chronic model of ICP and IOP modification.

## Conflict of Interests

None declared.

## Supporting Information

**Figure S1.** ICP during IOP elevation (mean ± SEM). ICP in groups of ICP = −5, 5, 25 and 30 mmHg were stable throughout the experiment (all slope <0.01, linear regression). All ICP was within 1.5 mmHg of target values.

**Figure S2.** Comparison of retinal surface position and thickness for different ICP levels at baseline IOP. At baseline IOP (10 mmHg) the effect of different ICP levels is examined. (A) There is no significant change for ONH (*P* = 0.551), central (*P* = 0.828), nor peripheral (*P* = 0.553), surface position. (B) no systematic ICP-induced change is found for central thickness, however due to the isolated finding at ICP = 25 mmHg of a thicker retina this returns *P* = 0.005. There is no significant change in peripheral retinal thickness (*P* = 0.345 and 0.838 respectively), retinal thickness. (C) No significant change is found in STR (*P* = 0.245) or b-wave function (*P* = 0.287).

**Figure S3.** Group average effect of IOP and ICP on surface position and retinal thickness. Data are shown as mean (±SEM) for three selected ICP levels as a function of IOP (low −5, normal 5 and high 30 mmHg) and distance from midline (*n* = 6–7). For clarity, data for ICP levels of 15 and 25 mmHg are not shown here. For each ICP level (mmHg) the IOP is denoted by warm to cool colors; 10 = red, 20 = orange, 30 = yellow, 40 = green, 60 = blue, 70 = purple. Surface position is shown in panels (A–C), whereas panels (D–F) show retinal thickness.

**Figure S4.** Effect of optic nerve pressure gradient modification on normalized retinal structure and function. Retinal thickness and electroretinogram parameters can be expressed relative to baseline IOP and thus normalized parameters are plotted against ONPG. Data are described using a two-line function where the intercept represents the critical ONPG threshold where effects become apparent. (A) thickness of the peripapillary retina (ONPG threshold = 14.8 mmHg; 95% CI: 3.1–28.8 mmHg). (B) Retinal ganglion cell-mediated (scotopic threshold response, STR) function (ONPG threshold = 50.6 mmHg; 95% CI: 38.7–58.7 mmHg). (C) bipolar cell-mediated (b-wave) function (ONPG threshold = 52.2 mmHg; 95% CI: 46.9–56.4 mmHg). Gray area indicates normal range of ONPG (0–15 mmHg).
