# Supplementary figures and images for: The effect of intraocular and intracranial pressure on retinal structure and function in rats
Source: Physiol Rep. 2015 Aug 19;3(8):e12507. doi: 10.14814/phy2.12507 (PMC4562590; doi:10.14814/phy2.12507)

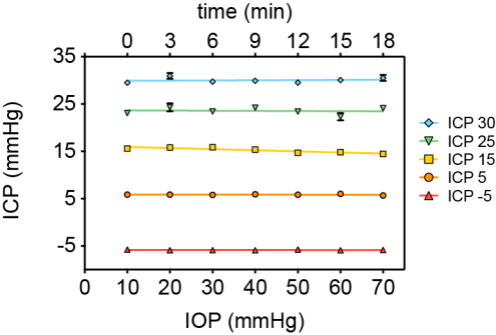

Supplement: Supplementary file 1 [file phy20003-e12507-sd1.pdf]

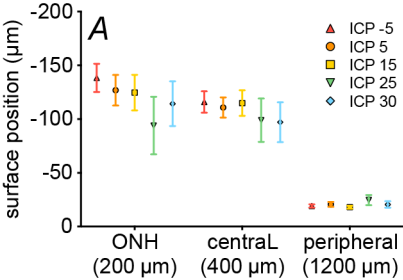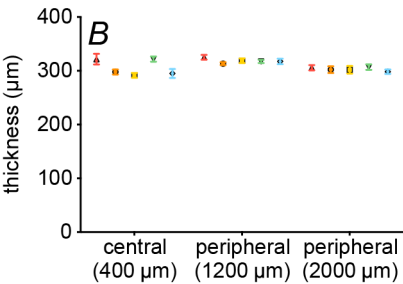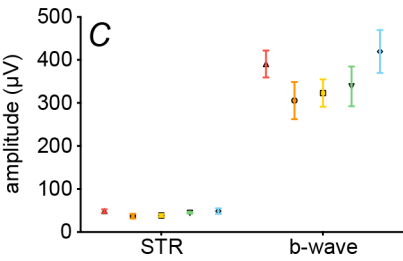

Supplement: Supplementary file 2 [file phy20003-e12507-sd2.pdf]

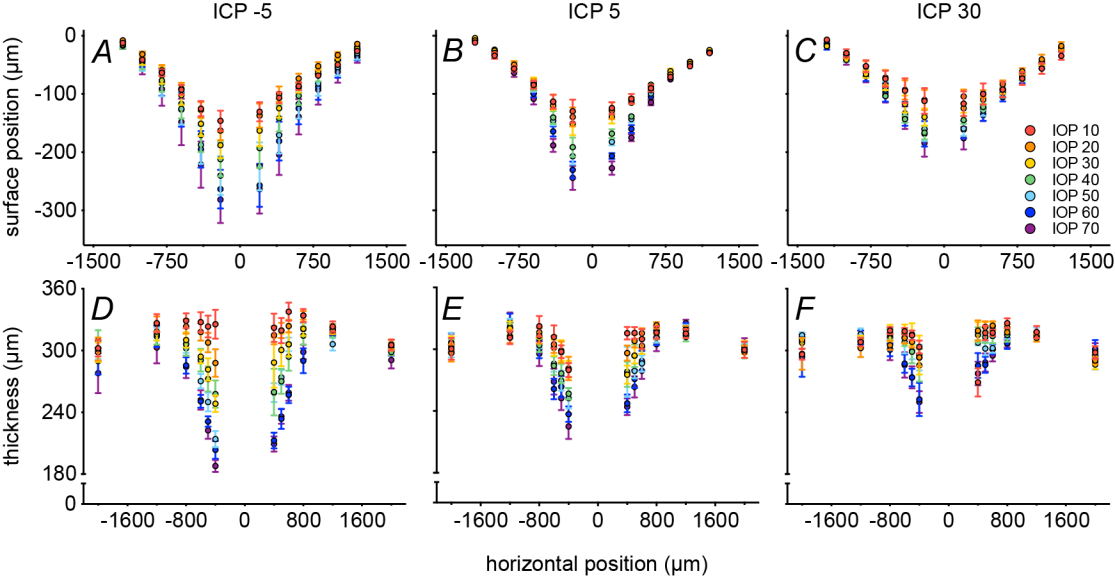

Supplement: Supplementary file 3 [file phy20003-e12507-sd3.pdf]

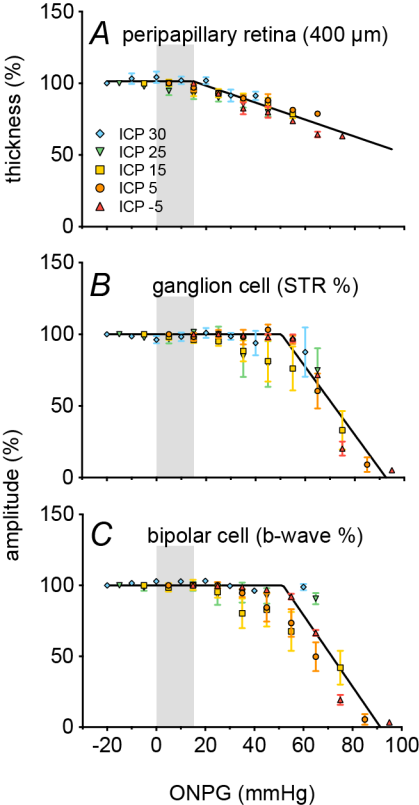

Supplement: Supplementary file 4 [file phy20003-e12507-sd4.pdf]
